# Japanese Multi‐Institution Study of Success Rates of Wire‐Guided Biliary Cannulation During Endoscopic Retrograde Cholangiopancreatography in Relation to Guidewire tip Length (With Video)

**DOI:** 10.1002/deo2.70144

**Published:** 2025-06-16

**Authors:** Takeshi Ogura, Yuki Tanisaka, Masanari Sekine, Katsumasa Kobayashi, Hirotsugu Maruyama, Shinji Hirai, Hideyuki Shiomi, Minoru Shigekawa, Masaki Kuwatani, Kenji Ikezawa, Masahiro Itonaga, Mamoru Takenaka, Susumu Hijioka, Tsukasa Ikeura, Shinpei Doi, Nao Fujimori, Kazuya Koizumi, Yousuke Nakai, Tadahisa Inoue, Shuntaro Mukai, Kazuyuki Matsumoto, Ryuki Minami, Koichiro Mandai, Atsuhiro Matsuda, Takuji Iwashita, Hiroki Kawashima, Takao Itoi

**Affiliations:** ^1^ Endoscopy Center Osaka Medical and Pharmaceutical University Osaka Japan; ^2^ Gastroenterology Saitama Medical University International Medical Center Hidaka Japan; ^3^ Department of Gastroenterology Jichi Medical University Saitama Medical Center Kawagoe Japan; ^4^ Department of Gastroenterology Tokyo Metropolitan Bokutoh Hospital Tokyo Japan; ^5^ Department of Gastroenterology Graduate School of Medicine Osaka Metropolitan University Osaka Japan; ^6^ Department of Medicine Division of Gastroenterology Kurume University School of Medicine Kurume Japan; ^7^ Department of Gastroenterology Division of Hepatobiliary and Pancreatic Diseases Hyogo Medical University Hyogo Japan; ^8^ Department of Gastroenterology and Hepatology Osaka University Graduate School of Medicine Osaka Japan; ^9^ Department of Gastroenterology and Hepatology Hokkaido University Hospital Hokkaido Japan; ^10^ Department of Hepatobiliary and Pancreatic Oncology, Osaka International Cancer Institute Osaka Japan; ^11^ Second Department of Internal Medicine, Wakayama Medical University Wakayama Japan; ^12^ Department of Gastroenterology and Hepatology Faculty of Medicine Kindai University Osaka Japan; ^13^ Department of Hepatobiliary and Pancreatic Oncology, National Cancer Center Hospital Tokyo Japan; ^14^ Third Department of Internal Medicine Kansai Medical University Hirakata Japan; ^15^ Department of Gastroenterology Teikyo University Mizonokuchi Hospital Kawasaki Japan; ^16^ Department of Medicine and Bioregulatory Science Graduate School of Medical Sciences Kyushu University Fukuoka Japan; ^17^ Department of Gastroenterology Medicine Center Shonan Kamakura General Hospital Kamakura Japan; ^18^ Department of Gastroenterology Graduate School of Medicine The University of Tokyo Tokyo Japan; ^19^ Department of Gastroenterology Aichi Medical University Nagakute Japan; ^20^ Department of Gastroenterology and Hepatology Tokyo Medical University Tokyo Japan; ^21^ Department of Gastroenterology and Hepatology, Okayama University Hospital Okayama Japan; ^22^ Department of Gastroenterology Tenri Hospital Nara Japan; ^23^ Department of Gastroenterology Kyoto Second Red Cross Hospital Kyoto Japan; ^24^ Department of Internal Medicine Toyama Prefectural Central Hospital Toyama Japan; ^25^ First Department of Internal Medicine Gifu University Hospital Gifu Japan; ^26^ Department of Gastroenterology and Hepatology Nagoya University Graduate School of Medicine Nagoya Japan

**Keywords:** ERCP, guidewire, pancreatitis, post‐ERCP pancreatitis, wire‐guided cannulation

## Abstract

**Objective:**

Wire‐guided cannulation (WGC) reportedly increases the successful biliary cannulation rate and reduces the risk of post‐endoscopic retrograde cholangiopancreatography pancreatitis. Currently, various types of guidewires are available. However, the effect of the length of flexible‐tip guidewires on the success rate of biliary cannulation under WGC and the rate of adverse events, especially post‐endoscopic retrograde cholangiopancreatography pancreatitis, is unclear. The aim of this study was to compare the influence of long‐tapered and short‐tapered tips of a 0.025‐inch guidewire on outcomes in primary selective biliary cannulation.

**Methods:**

Consecutive patients who underwent biliary access under endoscopic retrograde cholangiopancreatography guidance using WGC at 27 high‐volume centers in Japan were enrolled in this prospective registration study. The primary outcome was the technical success rate of biliary cannulation. The secondary outcomes were the rates of adverse events, biliary cannulation time, and number of guidewire insertions into the pancreatic duct.

**Results:**

A total of 530 patients underwent biliary cannulation for biliary disease with native papilla between April 2021 and December 2023. The technical success rate of biliary cannulation was 86.1% (161/187) in the long‐tip group and 84.3% (289/343) in the short‐tip group, indicating no significant differences between the two groups. Although the frequency of post‐endoscopic retrograde cholangiopancreatography was not significantly different, the successful biliary cannulation rate without guidewire mis‐insertion into the main pancreatic duct was significantly higher in the long tip group (64.7%, 121/187) compared with the short tip group (54.2%, 186/343*p* = 0.02).

**Conclusions:**

In conclusion, WGC using long‐tip guidewires might reduce the risk of guidewire insertion into the main pancreatic duct.

## 1 Introduction

Biliary cannulation under endoscopic retrograde cholangiopancreatography (ERCP) is the first step in diagnosing and treating biliary diseases. However, the failure rate of biliary cannulation using contrast medium injection with an ERCP catheter or the sphincterotome method reportedly ranges from 15 to 35% [1, 2]. In addition, the adverse event of post‐ERCP pancreatitis (PEP) is still an issue. Wire‐guided cannulation (WGC) reportedly increases the successful biliary cannulation rate and reduces the risk of PEP [3]. Therefore, not only the technique of guidewire manipulation but also appropriate guidewire selection during WGC is clinically important during biliary cannulation.

Currently, various types of guidewires are available. Regarding the diameter of the guidewire, 0.025‐ or 0.035‐inch guidewires are usually selected. Several comparative studies have shown shorter biliary cannulation time and easier device exchange with the 0.025‐inch guidewire compared with the 0.035‐inch guidewire [4, 5, 6]. Besides their size, guidewires also differ with respect to tip shape, flexibility, stiffness, and torque ability. According to several randomized controlled trials regarding WGC during biliary cannulation, the use of flexible‐tip [4] or curved‐tip type guidewires [7] improves successful biliary cannulation rates and decreases the rate of adverse events. On the other hand, the effect of the length of flexible‐tip guidewires on the success rate of biliary cannulation under WGC and the rate of adverse events, especially PEP, is unclear.

The aim of this study was to compare the influence of long‐tapered and short‐tapered tips of a 0.025‐inch guidewire on outcomes in primary selective biliary cannulation.

## 2 Methods

### 2.1 Patients

Consecutive patients who underwent biliary access under ERCP guidance using WGC at 27 high‐volume centers in Japan were enrolled in this retrospective study. The inclusion criteria were: (1) age >18 years, (2) presence of biliary disease, and (3) attempting biliary cannulation under WGC. The exclusion criteria were: (1) surgically altered anatomy, (2) attempting biliary cannulation using other techniques, (3) history of ERCP, and (4) refusal to participate in this study by the opt‐out method at each center.

### 2.2 Characteristics of the Guidewire and Biliary Cannulation Technique

**FIGURE 1:**
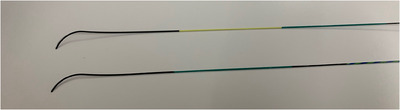
Kinds of guidewire: A long‐tapered tip (long type, above; J‐WIRE Prologue, J‐MIT Inc., Shiga, Japan) and a short‐tapered tip (short type, below; J‐WIRE Prologue ST, J‐MIT Inc.).

In this study, two types of 0.025‐inch guidewires were used during WGC: with a long‐tapered tip (long type; J‐WIRE Prologue; J‐MIT Inc., Shiga, Japan) or a short‐tapered tip (short type; J‐WIRE Prologue ST; J‐MIT Inc.) (Figure 1). The guidewire tips are coated with polyurethane, and the shaft is mainly coated with polytetrafluoroethylene. The core wire is included in the external coating and gradually tapers toward the tip. A spiral‐coiled spring is present around the tip of the core wire. The core wire material consists of nickel‐titanium (Ni‐Ti). The length of the tip of the long type is 140 mm, and of the short type is 100 mm.

**FIGURE 2:**
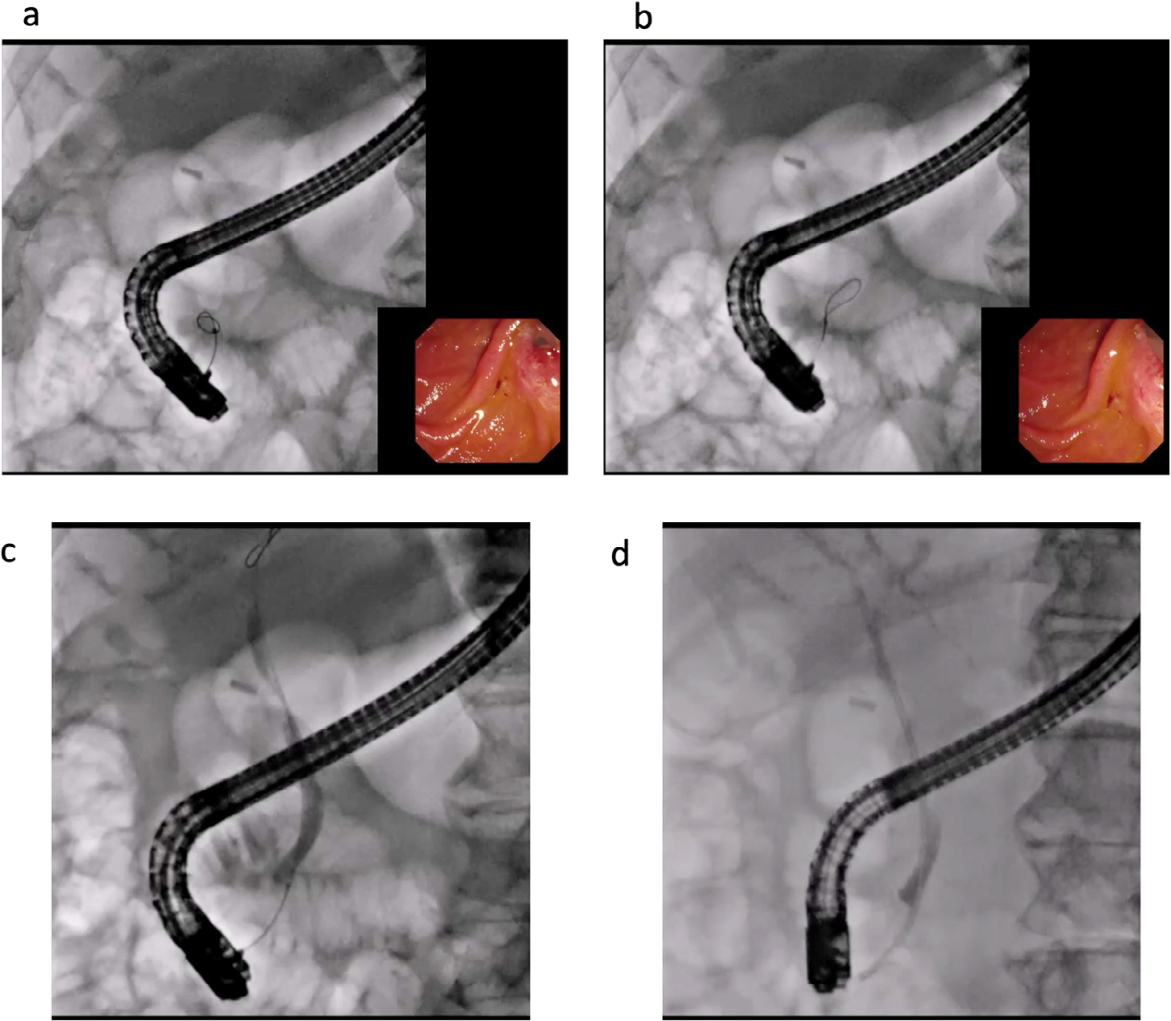
Biliary cannulation using long‐type guidewire: (a) Biliary cannulation is performed, but the guidewire is looped within the bile duct. (b) Using pushing guidewire, guidewire can be advanced into the biliary tract with a loop shape. (c) The contrast medium is injected. (d) Biliary stenting is performed.

Technical tips for biliary cannulation are shown in Figure 2. Briefly, the duodenoscope (TJF or JF 260; Olympus Medical Tokyo, Japan and ED‐580XT; Fujifilm, Tokyo, Japan) is inserted into the duodenum, and the ampulla of Vater is visualized. Then, biliary cannulation is attempted using a standard ERCP catheter combined with WGC (Figure 2a,b). WGC in this study was defined as the spearhead of the ERCP catheter being contacted by the ampulla of Vater, and guidewire manipulation was performed by the operator or the assistant doctor. If the guidewire is accidentally inserted into the pancreatic duct, it is withdrawn, and biliary cannulation is performed again. The contrast medium is only injected after successful biliary cannulation (Figure 2c). After successful guidewire insertion into the bile duct, the required procedures, such as stone removal, stent deployment, or biopsy, are performed (Figure 2d; Video S1).

### 2.3 Outcome Measurements and Definitions

The primary outcome was the technical success rate of biliary cannulation. Successful biliary cannulation was defined as ERCP catheter insertion into the biliary tract using WGC. If biliary cannulation was performed using WGC combined with other techniques, such as the double guidewire technique, the case was considered a technical failure. The secondary outcomes were the rates of adverse events, biliary cannulation time, and number of guidewire insertions into the pancreatic duct. Adverse events were graded according to the severity grading system of the American Society for Gastrointestinal Endoscopy lexicon [8]. Procedure time was measured from the start of biliary cannulation to successful biliary cannulation. In this study, if the number of ERCPs performed by the endoscopist was over 100 cases, the endoscopist was considered an expert. Several factors, such as technical success, procedure time, and number of guidewire insertions into the pancreatic duct were evaluated using videos recorded at each center. Descriptive statistics are presented as the mean ± standard deviation (SD) for continuous variables, and as frequencies for categorical variables. The two study arms were compared using analysis of variance for continuous factors, the Kruskal–Wallis test for number of events, and Pearson's chi‐square test or Fisher's exact test for categorical factors. All data were statistically analyzed using SPSS version 13.0 statistical software (SPSS, Chicago, IL; Table 1).

**TABLE 1:** Patients’ characteristics and clinical outcomes.

	Long‐tip type (*n* = 187)	Short‐tip type (*n* = 343)	*p*‐value
Age (median, years)	72.7 ± 12.3	71.6 ± 12.0	0.55
Sex (male:female)	86:101	185:158	0.08
Disease, *n*			0.23
Biliary stricture	84	181	
Bile duct stones	76	122	
Pancreatic duct stricture	17	29	
Others	10	11	
Operator, *n* (expert:non‐expert)	62:125	131:212	0.26
Procedure time (≥10 min:<10 min)	130:57	229:114	0.56
Successful biliary cannulation rate without guidewire mis‐insertion into the main pancreatic duct, % (*n*)	64.7 (121/187)	54.2 (186/343)	0.02
Success rate of PCGW, % (*n*)	86.1% (161/187)	84.3% (289/343)	0.61
Post‐ERCP pancreatitis, % (*n*)	4.5 (8/187)	3.2 (11/343)	0.63

## 3 Results

A total of 530 patients underwent biliary cannulation for biliary disease with native papilla between April 2021 and December 2023. Among them, biliary cannulation was performed using the long tip guidewire in 187 patients (mean age, 72.7 years; 86 males, 101 females), and short type tip in 343 patients (mean age, 71.6 years; 185 males, 158 females). In both groups, the main indication for ERCP was biliary stricture or bile duct stone removal, and these indications were not significantly different between the two groups (*p* = 0.23). Biliary cannulation was performed by 62 experts and 125 non‐experts in the long tip group, and by 131 experts and 212 non‐experts in the short tip group, with no significant differences (*p* = 0.26).

The technical success rate of biliary cannulation was 86.1% (161/187) in the long‐tip group and 84.3% (289/343) in the short‐tip group, indicating no significant differences between the two groups (*p* = 0.61). Additionally, the number of patients who underwent successful biliary cannulation over 10 min was 57 in the long‐tip group and 114 in the short‐tip group, indicating no significant difference between them (*p* = 0.56). Although the frequency of PEP was not significantly different between the long (4.5%, 8/187) and short tip (3.2, 11/343) groups (*p* = 0.63), the successful biliary cannulation rate without guidewire mis‐insertion into the main pancreatic duct was significantly higher in the long tip group (64.7%, 121/187) compared with the short tip group (54.2%, 186/343; *p* = 0.02).

## 4 Discussion

During biliary cannulation, consideration should be given to improving the technical success rate and decreasing the frequency of PEP. To this end, various efforts have been reported to date [3, 4, 5, 6, 7, 9, 10, 11, 12, 13, 14, 15]. Among the reported techniques, WGC might improve the technical success rate of biliary cannulation, possibly also reducing PEP [3, 15, 16, 17]. According to a meta‐analysis of WGC that included 12 randomized controlled trials [15], WGC significantly reduced PEP compared with the contrast‐assisted cannulation technique (risk ratio [RR] 0.51, 95% confidence interval [CI] 0.32–0.82). In addition, WGC is associated with greater primary cannulation success (RR 1.07, 95% CI 1.00–1.15), fewer precut sphincterotomies (RR 0.75, 95% CI 0.60–0.95), and no increase in other ERCP‐related complications. The characteristics of the guidewire, however, play a central role during WGC.

Regarding the diameter of the guidewire, such as 0.025 or 0.035 inches, the successful biliary cannulation rate with WGC was similar between the 0.025‐inch and 0.035‐inch wire groups (80.7% vs. 80.3%; *p* = 0.90). In a previous randomized controlled trial, the incidence of PEP between the 0.025‐inch and 0.035‐inch wire groups was not significantly different (7.8% vs 9.3%; *p* = 0.51) [5]. However, the 0.025‐inch guidewire might have theoretical benefits, such as less bile duct or pancreatic duct orifice injury, and easy access to the biliary tract due to the small caliber of the guidewire, since it enables negotiation of the common channel and crossing of the intrapapillary septum [4, 5, 6]. Therefore, in our study, a 0.025‐inch guidewire was selected.

The shape of the guidewire tip might also play an important role during WGC. Vihervaara et al. conducted a randomized controlled trial including 153 patients who were included in angled‐ (*n* = 70) or straight‐tipped guidewire groups (*n* = 83) for WGC [18]. In their study, although the primary cannulation success rate and rate of adverse events were not significantly different between the two groups, the median successful cannulation time was significantly shorter in the angled‐tipped guidewire group (20 min) compared with the straight‐tipped guidewire group (44 min; *p* = 0.01). Maki et al. evaluated these guidewire types during WGC in non‐expert hands [19]. They observed a significantly higher selective biliary cannulation success rate over 14 min with an angled‐tipped guidewire compared with a straight‐tipped guidewire (57.8% vs. 34.3%, *p* = 0.04), and with success rates of 36.4% and 0%, respectively (*p* = 0.04), within 21 min. On the other hand, Tsuchiya et al. conducted a multicenter, controlled study comparing an angle‐tipped guidewire with the J‐tip guidewire, the angle of which is more acute compared with that of the angle‐tipped guidewire [20]. The rate of successful biliary cannulation within 10 min was 84.8% (55/66) with the J‐tip and 80.0% (52/65) with the angle‐tipped guidewire, and the incidence rates of complications were 3.0% (2/66) and 6.2% (4/65) in the J‐tip and angle‐tipped guidewire groups, respectively. There were no significant differences in any results between both groups. Therefore, since the J‐tip guidewire might not have clinical benefits during WGC, we selected an angle‐tipped guidewire for our study.

Flexibility of the guidewire might also play an important role during WGC. Park et al. conducted a randomized trial of WGC comparing a flexible guidewire and a conventional guidewire [4]. In their study, 100 patients were divided into flexible guidewire (*n* = 50) and conventional guidewire groups (*n* = 50). The success rate of biliary cannulation using WGC was higher in the flexible guidewire group (96%, 48/50) compared with the conventional guidewire group (86%, 43/50; *p* = 0.08). Mean biliary cannulation time was significantly shorter in the flexible guidewire group (174.9 s) compared with the conventional guidewire group (363.5 s; *p* = 0.04). Therefore, as noted above, a 0.025‐inch, angle‐tipped, flexible guidewire might be suitable for the successful performance of WGC. However, the role of the length of the tip of the guidewire during WGC has not been previously evaluated. The present study might be the first report from the viewpoint of the length of the guidewire tip.

**FIGURE 3:**
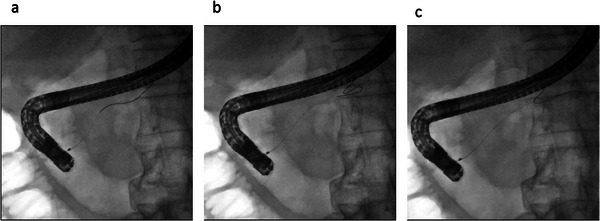
Pancreatic duct cannulation using long‐type guidewire: (a) Guidewire is inserted into the main pancreatic duct. (b) Using pushing guidewire, a loop shape can be automatically made. (c) Guidewire advancement is successfully performed without duct penetration.

During biliary cannulation, the guidewire will be returned when the tip contacts a wall, forming a loop. Our previous experimental study showed that the loop shape is easily formed if the tip length is long, although other factors, such as shaft stiffness or flexibility, also influence the looping of the guidewire [21]. The loop shape is useful in several ways during ERCP. First, duct penetration is prevented by the guidewire curling into a loop shape. In addition, during biliary cannulation, the loop shape might facilitate the passage of the guidewire through the epithelial folds of the intra‐duodenal biliary segments. Theoretically, this would increase the success rate of WGC. On the other hand, if the loop does not form, the guidewire might penetrate the epithelial folds, increasing the risk of papillary trauma during WGC [20]. In addition, according to several studies [20, 21], the loop shape prevents the risk of insertion of the guidewire into the pancreatic duct. Indeed, in the present study, since the J‐WIRE prologue is angle‐tipped and flexible with a long tip, there were fewer instances of guidewire insertion into the main pancreatic duct compared with the short‐type tip. Therefore, from a safety perspective, long‐tip guidewires might be useful during biliary cannulation. Indeed, if the long tip guidewire is inserted into the main pancreatic duct branch, it leads to loop formation, thereby preventing duct penetration (Figure 3a–c). Additionally, during biliary cannulation, bile duct penetration might also be prevented by looping the guidewire. In the present study, although data on the number of cases in which the guidewire formed a loop is lacking, long‐tip guidewires might be safe during WGC.

The present study has multiple critical limitations. First, although the presented study included a large number of centers, it was non‐randomized and retrospective in nature. Second, various important data, such as the shape of the ampulla of Vater, laboratory data, and the patient's background characteristics were missing. This fact is a serious limitation because we cannot evaluate the role of different types of guidewires for PEP. Third, there are various factors to consider when selecting a guidewire, therefore, it is unclear whether the present study, which examined a guidewire from a specific manufacturer, can conclude that the log tip is superior. However, our study might be the first study investigating the role of differences in guidewire tip length during WGC, making it a landmark for further trials. A prospective randomized trial will be conducted by our group.

In conclusion, WGC using long‐tip guidewires might reduce the risk of guidewire insertion into the main pancreatic duct, without decreasing the success rate of biliary cannulation compared with short‐tip guidewires. Further randomized trials are needed to confirm our results.

## Conflicts of Interest

Takeshi Ogura and Shuntaro Mukai are associated Editors of DEN Open. Takao Itoi is the Editor‐in‐Chief of DEN Open. The other authors declare no conflicts of interest.

## Ethics Statement

Approval of the research protocol by an Institutional Reviewer Board (IRB No: 2024‐015).

## Consent

N/A

## Clinical Trial Registration

N/A

## Supporting information



wire‐guided cannulation using long‐tapered tip guidewire .mp4
